# Snake Toxins Labeled by Green Fluorescent Protein or Its Synthetic Chromophore are New Probes for Nicotinic acetylcholine Receptors

**DOI:** 10.3389/fmolb.2021.753283

**Published:** 2021-11-30

**Authors:** Igor E. Kasheverov, Alexey I. Kuzmenkov, Denis S. Kudryavtsev, Ivan S. Chudetskiy, Irina V. Shelukhina, Evgeny P. Barykin, Igor A. Ivanov, Andrei E. Siniavin, Rustam H. Ziganshin, Mikhail S. Baranov, Victor I. Tsetlin, Alexander A. Vassilevski, Yuri N. Utkin

**Affiliations:** ^1^ Shemyakin-Ovchinnikov Institute of Bioorganic Chemistry, Russian Academy of Sciences, Moscow, Russia; ^2^ Engelhardt Institute of Molecular Biology, Russian Academy of Sciences, Moscow, Russia; ^3^ Moscow Institute of Physics and Technology, Moscow Region, Russia

**Keywords:** nicotinic acetylcholine receptor, acetylcholine-binding protein, α-cobratoxin, azemiopsin, fluorescent protein, fluorescent toxin, ion channel, peptide toxin

## Abstract

Fluorescence can be exploited to monitor intermolecular interactions in real time and at a resolution up to a single molecule. It is a method of choice to study ligand-receptor interactions. However, at least one of the interacting molecules should possess good fluorescence characteristics, which can be achieved by the introduction of a fluorescent label. Gene constructs with green fluorescent protein (GFP) are widely used to follow the expression of the respective fusion proteins and monitor their function. Recently, a small synthetic analogue of GFP chromophore (*p*-HOBDI-BF_2_) was successfully used for tagging DNA molecules, so we decided to test its applicability as a potential fluorescent label for proteins and peptides. This was done on α-cobratoxin (α-CbTx), a three-finger protein used as a molecular marker of muscle-type, neuronal α7 and α9/α10 nicotinic acetylcholine receptors (nAChRs), as well as on azemiopsin, a linear peptide neurotoxin selectively inhibiting muscle-type nAChRs. An activated N-hydroxysuccinimide ester of *p*-HOBDI-BF_2_ was prepared and utilized for toxin labeling. For comparison we used a recombinant α-CbTx fused with a full-length GFP prepared by expression of a chimeric gene. The structure of modified toxins was confirmed by mass spectrometry and their activity was characterized by competition with iodinated α-bungarotoxin in radioligand assay with respective receptor preparations, as well as by thermophoresis. With the tested protein and peptide neurotoxins, introduction of the synthetic GFP chromophore induced considerably lower decrease in their affinity for the receptors as compared with full-length GFP attachment. The obtained fluorescent derivatives were used for nAChR visualization in tissue slices and cell cultures.

## Introduction

Three-finger toxins (TFTs) are common widespread non-enzymatic components of elapid snake venoms. TFTs comprise ∼60–80 amino acid residues folded into three β-hairpin loops extending from a hydrophobic core (Kini and Doley, 2010; Kini and Koh, 2020). The name “three-finger toxins” originates from these three loops resembling three fingers of the human hand. Usually, these toxins are stabilized by four or five disulfide bridges, four of which are conserved (Kessler et al., 2017). Among these polypeptides there are well-known ligands of several ion channels including α-neurotoxins (nicotinic acetylcholine receptors, nAChRs) (Barber et al., 2013), Tx7335 (voltage-gated potassium channels) (Rivera-Torres et al., 2016), calliotoxin (sodium channels) (Yang et al., 2016), calciseptine (calcium channels) (de Weille et al., 1991), mambalgins (acid-sensing ion channels) (Diochot et al., 2012), and micrurotoxins (receptors of γ-aminobutyric acid) (Rosso et al., 2015). TFTs represent one of the best studied natural combinatorial libraries with immense potential for drug discovery.

The history of TFTs began with the discovery of α-bungarotoxin (α-BgTx) in the venom of the many-banded krait *Bungarus multicinctus* about 60 years ago (Chang and Lee, 1963). Dozens of TFTs, such as α-cobratoxin (α-CbTx) from the monocled cobra *Naja kaouthia* (Karlsson et al., 1972), neurotoxin NTII from the Caspian cobra *Naja oxiana*, and LsIII from the black-banded sea krait *Laticauda semifasciata*, were further purified and characterized (Utkin, 2019; Tsetlin et al., 2021). Those toxins served as high-affinity and selective molecular probes *per se*, and their labeled derivatives are traditionally utilized as robust molecular reporter tools in channel research (Anderson and Cohen, 1974; Stiles, 1993; Kuzmenkov and Vassilevski, 2018; Kudryavtsev et al., 2020). Pioneer works, where tritiated and iodinated α-BgTx was used, introduced the methodology of nAChR purification, identification of binding sites, and determination of the receptors distribution in the brain (O’Brien et al., 1972; Silver and Billiar, 1976; Lukasiewicz and Bennett, 1978). TFTs with isotopic moieties are still widely used in radioligand binding assays for the detection of novel compounds and establishing their kinetic properties (Martin et al., 2007; Anderson, 2008). An alternative approach to develop toxin-based molecular markers is the utilization of fluorescent dyes. Fluorescent derivatives of α-BgTx were used for visualizing the distribution of nAChR in vertebrate skeletal muscle fibers, expression profiling and description of the clusterization process of diffusely distributed receptors (Ravdin and Axelrod, 1977; Stya and Axelrod, 1983; Anderson, 2008; Shelukhina et al., 2009). Simultaneously, fluorescently-modified NTII and α-CbTx were also successfully produced and applied for binding studies and mapping of nAChR (Tsetlin et al., 1979, 1982; Kang and Maelicke, 1980).

Here, we extend the techniques of toxin labeling by the design and production of novel fluorescent probes for nAChR imaging. For these purposes we obtained three derivatives of well-studied toxins. The first marker is the recombinant protein eGFP–α-CbTx, which is a fusion of α-CbTx and the enhanced green fluorescent protein (eGFP). α-CbTx binds with high affinity and practically irreversibly to nAChRs; therefore, even after some decrease in affinity as a result of modification this toxin retains the capacity to bind to its receptor. Earlier, it was shown that attachment of a fluorescein label at Lys23 in the central loop of α-CbTx resulted in a fluorescently labeled derivative retaining nanomolar affinity to *Torpedo* nAChR (Johnson and Taylor, 1982). For quite a long time we have been using α-CbTx and its derivatives for the study of different receptors (Utkin et al., 1998; Kudryavtsev et al., 2015) and gained extensive experience in work with just this toxin. Moreover, we possess sufficient quantities of native α-CbTx for comparison. All of this contributed to our decision to choose α-CbTx for preparation of chimera with eGFP. We have already successfully utilized conjugation with eGFP to produce and characterize selective probes to voltage-gated potassium channels (Kuzmenkov et al., 2016a). Such method of labeling is aimed at obtaining mono-tagged derivatives and claims to dramatically decrease the costs of the final products (Kuzmenkov and Vassilevski, 2018). The second fluorescent probe is presented by the same neurotoxin covalently linked to eGFP chromophore-mimicking moiety (*p*-HOBDI-BF_2_) (Baranov et al., 2012). This tag was previously applied only for labeling DNA molecules (Stakheev et al., 2018) and used in the preparation of an inhibitor for irreversible interactions with the active-site cysteine of human cathepsins (Frizler et al., 2013). Moreover, to design specific muscle-type nAChR reporter we utilized *p*-HOBDI-BF_2_ moiety for the preparation of a mono-labeled fluorescent derivative of azemiopsin (Aze), a linear neurotoxic peptide earlier isolated from the *Azemiops feae* viper venom (Utkin et al., 2012).

## Material and Methods

### Materials

α-CbTx was purified from *N. kaouthia* venom as described (Osipov et al., 2008). Aze was synthesized as in (Shelukhina et al., 2018). Radiolabeled ^125^I-α-BgTx with specific radioactivity of 500 Ci/mmol was prepared as in (Kryukova et al., 2018). Alexa Fluor 555 and 647 conjugates of α-BgTx (AF555–α-BgTx and AF647–α-BgTx) were purchased from Thermo Fisher Scientific.

Chymotrypsin was from Sigma and Lys-C protease was from BDH Chemicals (Sweden). Recombinant human enteropeptidase light chain was a gift from Dr. Marine Gasparian (Gasparian et al., 2003). Acetonitrile was from PanReac-AppliChem (ITW Reagents, Spain). Commercially available reagents were used for activated *p*-HOBDI-BF_2_ synthesis without additional purification. All other reagents were of analytical or higher grade.

To construct an expression vector bearing the eGFP–α-CbTx gene, we used pET-28a (Novagen). The gene of α-CbTx including a stop codon was synthesized by Evrogen (Russia).

Murine neuroblastoma Neuro-2a cells were obtained from ATCC-LGC (France) and maintained as described previously (Shelukhina et al., 2017). THP-1 (ATCC TIB-202), Raji (ATCC CCL-86), and RPMI 1788 (ATCC CCL-156) cells were maintained in RPMI 1640 medium, supplemented with 10% fetal bovine serum, 1× GlutaMAX and 1× penicillin-streptomycin solution (all from Gibco, Thermo Fisher Scientific) at 37°C with 5% CO_2_.

### Construction of a Vector Harboring the eGFP–α-CbTx Gene

At the first step the pET-28a-eGFP vector was obtained by cloning the eGFP gene alone, which was amplified with eGFP-f and eGFP-r primers from the plasmid pUC-eGFP, as we described previously (Kuzmenkov et al., 2016a) (Table 1). NdeI and BamHI sites were used for cloning. On the second step, a sequence encoding a short linker was synthesized from primers (Link-f and Link-r) and added at BamHI and EcoRI restriction sites. Finally, the gene of α-CbTx with a stop codon was amplified with CbTx-f and CbTx-r oligonucleotides and then subcloned in pET-28a-eGFP using EcoRI and SalI restriction sites. Additionally, α-CbTx gene was produced with modified human enteropeptidase site (DDDDR) at the N-terminus by changing CbTx-f to CbTx-f-EK primer. All oligonucleotides from which the full genes encoding eGFP–α-CbTx and eGFP–EK-α-CbTx were generated by PCR amplification are listed in Table 1. Correct cloning of the genes was confirmed by sequencing.

**TABLE 1:** List of oligonucleotides used for eGFP–α-CbTx and eGFP–EK-α-CbTx gene construction.

Oligonucleotide name	Sequence
eGFP-f	5′ CAC​GCA​TAT​GAT​GGT​GAG​CAA​GGG​C 3′
eGFP-r	5′ ATA​TGG​ATC​CCT​CGT​ACA​GCT​CGT​CC 3′
Link-f	5′ ATA​TGG​ATC​CGG​AGG​CGG​TGG​CTC​GGG​AGG​TGG​CGG​TTC​GG 3′
Link-r	5′ TAT​AGA​ATT​CGG​AGC​CGC​CAC​CGC​CCG​AAC​CGC​CAC​CTC​CC 3′
CbTx-f	5′ GCA​TGA​ATT​CAT​CCG​CTG​CTT​CAT​CAC​CCC​A 3′
CbTx-f-EK	5′ATA​TGA​ATT​CGA​TGA​TGA​TGA​TCG​TAT​CCG​CTG​CTT​CAT​CAC​CCC​AGA​CAT​TAC​G 3′
CbTx-r	5′ ATA​TGT​CGA​CTT​ACG​GAC​GTT​TGC​GGG​TTG​GAA​A 3′

### Production and Purification of eGFP–α-CbTx and eGFP–EK-α-CbTx

*Escherichia coli* BL21 (DE3) cells transformed with pET-28a-eGFP–α-CbTx and pET-28a-eGFP–EK-α-CbTx were cultured at 37°C in LB medium in the presence of 50 ng/ml kanamycin to the mid-log phase. Expression of the fluorescent chimera gene was induced by 0.5 mM IPTG and the culture was further incubated at 25°C for 20 h. Cells were harvested by centrifugation, disrupted by sonication, and the chimeric proteins were purified from the soluble fraction by affinity chromatography on a TALON Superflow resin (Clontech) following the manufacturer’s protocol. Further purification was performed by size-exclusion chromatography on a TSK 2000SW column (7.5 × 600 mm, 12.5 nm pore size, 10 μm particle size; Toyo Soda Manufacturing) in phosphate-buffered saline (PBS), pH 7.4, at a flow rate of 0.5 ml/min. Expression and purification of the target proteins was monitored by SDS-PAGE. Concentration of the final eGFP–α-CbTx and eGFP–EK-α-CbTx preparations was measured by absorption spectroscopy using ε (489 nm) = 55,000 M^−1^cm^−1^.

### eGFP–EK-α-CbTx Digestion by Human Enteropeptidase Light Chain

eGFP–EK-α-CbTx chimera produced in bacteria was subjected to treatment by enteropeptidase. The recombinant protein was dissolved in 50 mM Tris-HCl (pH 8.0) to a concentration of 1 mg/ml. Protein cleavage with human enteropeptidase light chain (1 U of enzyme per 1 mg of substrate) was performed overnight (16 h) at 37°C. The mixture was separated by reversed-phase HPLC on a Jupiter C5 column (4.6 × 250 mm; Phenomenex) in a linear gradient of acetonitrile concentration (0–60% in 60 min) in the presence of 0.1% trifluoroacetic acid, at a flow rate of 1 ml/min. Purified recombinant α-CbTx was assessed by MALDI mass-spectrometry.

### Mass Spectrometry

Ultraflex TOF-TOF (Bruker Daltonik, Germany) spectrometer was used for MALDI mass spectrometry as described previously (Kuzmenkov et al., 2016b). 2,5-Dihydroxybenzoic acid (Sigma-Aldrich) was used as a matrix. Measurements were performed in the linear mode. Mass spectra were analyzed with the Data Analysis 4.3 and Data Analysis Viewer 4.3 software (Bruker).

High-resolution mass spectra of activated *p*-HOBDI-BF_2_ were recorded on a TripleTOF 5600+ System (SCIEX) using electrospray ionization (ESI). The measurements were done in a positive ion mode (interface capillary voltage of 5500 V); mass range from m/z 50 to m/z 3,000; external or internal calibration was done with the ESI Tuning Mix (Agilent). A syringe injection was used for solutions in acetonitrile, methanol, or water (flow rate of 20 μl/min). Nitrogen was applied as a dry gas; interface temperature was set at 180°C.

ESI mass spectra of Aze fragments were recorded on an LTQ Orbitrap XL instrument (Thermo Fisher Scientific) equipped with HESI-II ion source. The acquisition was performed using full-scan FTMS mode at 30K resolution in a positive ions detection mode within the 700–2000 mass range.

### Synthesis of (Z)-2,5-Dioxopyrrolidin-1-yl 4-(4-(2-(difluoroboryl)-4-hydroxybenzylidene)-2-methyl-5-oxo-4,5-dihydro-1H-imidazol-1-yl)butanoate (*p*-HOBDI-BF_2_-OSu)

Kieselgel 60 (Merck, Germany) was used for column chromatography. Thin-layer chromatography was performed on silica gel 60 F_254_ glass-backed plates (Merck). Visualization was performed using UV light (254 or 312 nm) and staining with KMnO_4_. NMR spectra were recorded on a 700 MHz Bruker Avance III instrument at 303 K. Chemical shifts are reported relative to residual peaks of DMSO-d_6_ (2.51 ppm for ^1^H and 39.5 ppm for ^13^C). Melting points were measured on a Stuart SMP30 apparatus (Cole-Parmer) without correction. IUPAC compound names were generated using ChemDraw software (PerkinElmer).

1.0 mmol of (Z)-4-(4-(2-(difluoroboryl)-4-hydroxybenzylidene)-2-methyl-5-oxo-4,5-dihydro-1H-imidazol-1-yl)butanoic acid (Frizler et al., 2013) was dissolved in dry THF (30 ml), and N,N,N′,N′-tetramethyl-O-(N-succinimidyl)uronium tetrafluoroborate (330 mg, 1.1 mmol) and triethylamine (120 mg, 1.2 mmol) were added. The mixture was stirred at room temperature for 1 h. Hydrofluoric acid (water solution, 48%, 0.5 ml) was added and the mixture was stirred for additional 10 min. The mixture was dissolved by EtOAc (150 ml), washed by brine (3 × 30 ml), dried over Na_2_SO_4_, and evaporated. The product (N-hydroxysuccinimide ester of *p*-HOBDI-BF_2_, *p*-HOBDI-BF_2_-OSu) was purified by flash chromatography (using EtOAc:THF = 2:1 mixture as an eluent): yellow solid, 370 mg (85%), m. p. = 163–165°С.

^1^H NMR (DMSO-d_6_) δ 10.19 (s, 1H), 7.55 (s, 1H), 7.49 (d, J = 8.1Hz, 1H), 7.00 (d, J = 2.5Hz, 1H), 6.74 (dd, J_1_ = 8.2Hz, J_2_ = 2.5Hz, 1H), 3.80 (t, J = 7.27Hz, 2H), 2.85 (t, J = 7.48Hz, 2H), 2.82 (br.s, 4H), 2.74 (s, 3H), 1.99 (p, J = 7.48Hz, 2H).

^13^C NMR (DMSO-d_6_) δ 12.65 (CH_3_), 22.90 (CH_2_), 25.40 (2×CH_2_), 27.52, 39.88, 115.12 (CH), 118.48 (CH), 124.00, 124.87, 129.25 (CH), 134.27 (CH), 161.41, 162.93, 164.18, 168.51, 170.11.

High resolution mass spectrometry (ESI) [M + H]^+^ m/z: 434.1341 found; 434.1335 calculated for C_19_H_19_BF_2_N_3_O_6_.

### Modification of α-CbTx and Aze with *p-*HOBDI-BF_2_

α-CbTx was modified with *p*-HOBDI-BF_2_-OSu and the derivatives were purified exactly under conditions used earlier for the preparation of α-CbTx mono-labeled derivatives (Utkin et al., 1998). To obtain Aze derivatives, 5.3 mg of Aze were dissolved in 1 ml of 0.2 M sodium phosphate buffer (pH 8.2) and 1.1 mg of *p*-HOBDI-BF_2_-OSu in 100 µl of dioxane was added to the toxin solution. The mixture was incubated overnight at room temperature and desalted on a Sephadex G-10 column (1 × 90 cm) equilibrated in 0.1 M acetic acid. After freeze-drying the mixture was separated by reversed-phase HPLC on a Jupiter C_18_ column (10 × 250 mm; Phenomenex) in a linear gradient of acetonitrile (15–30% in 75 min) in the presence of 0.1% trifluoroacetic acid, at a flow rate of 2 ml/min. After freeze-drying, the obtained derivatives (HOBDI-BF_2_–Aze) were used for further studies.

### Localization of Label with Chromato-Mass Spectrometry Analysis

Purified HOBDI-BF_2_–Aze conjugate was dissolved in 50 mM ammonium bicarbonate solution (pH 9.0) to a final concentration of 1 mg/ml, then subjected to digestion by chymotrypsin or Lys-C protease (1 : 25, enzyme: substrate, w/w) at 37°C for 24 h, followed by formic acid quenching (1% by vol). The digested samples were analyzed by LC-MS on a YMC-Triart C18 column (2.1 × 150 mm; YMC) in a linear gradient of acetonitrile (5–55%) at flow rate of 0.3 ml/min. The modified peptide fragments were detected by specific dye absorption at 415 nm.

### Competitive Radioligand Assay

In competition experiments with ^125^I-α-BgTx (500 Ci/mmol), all tested compounds (α-CbTx, HOBDI-BF_2_–α-CbTx, eGFP–α-CbTx, eGFP, Aze, and HOBDI-BF_2_–Aze derivatives) at different concentrations were pre-incubated for 2.5 h at room temperature with the great pond snail *Lymnaea stagnalis* acetylcholine binding protein (AChBP) (at the final concentration of 2.5 nM), or with GH_4_C_1_ cells (final concentration of 0.4 nM of toxin-binding sites), or *Torpedo californica* electric organ membranes (final concentration of 0.5 nM of toxin-binding sites) in 50 μL of binding buffer (20 mM Tris-HCl, 1 mg/ml bovine serum albumin (BSA), pH 7.5). After that ^125^I-α-BgTx was added to AChBP, GH_4_C_1_ cells or membranes to a final concentration of 0.5 nM, and the mixtures were additionally incubated for 5 min. Binding was stopped by rapid filtration on double DE-81 filters (Whatman) pre-soaked in the binding buffer (for AChBP) or on GF/C filters (Whatman) pre-soaked in 0.25% polyethylenimine (for GH_4_C_1_ cells or membranes), unbound radioactivity being removed from the filters by washout (3 × 3 ml) with the binding buffer. Non-specific binding was determined in all cases using 2.5 h pre-incubation with 10 μM α-CbTx. The binding results were analyzed using OriginPro 2015 program (OriginLab Corporation) fitting to a one-site dose-response curve with the following equation: $\%response=100/\left\{1+{\left(\left[toxin\right]/IC_{50}\right)}^{n_{H}}\right\}$ , where *n*_*H*_ is the slope factor (Hill coefficient). Data are presented as means ± standard errors of means (S.E.M.).

### Thermophoresis

For the microscale thermophoresis (MST) experiments, both the fluorescent ligands (HOBDI-BF_2_–α-CbTx or eGFP–α-CbTx) and AChBP were diluted in PBS, pH 7.2, containing 0.05% Tween. Then, glass capillaries (NanoTemper Technologies, Germany) were filled with different concentrations of AChBP and with the same concentration of the fluorescent ligand. The MST experiments were performed with Monolith NT.115 (NanoTemper Technologies, Germany) according to the manufacturer’s recommendations. Before each experiment, a pretest was carried out to check the fluorophore’s stability and exclude the sorption of the fluorescent molecule on the capillary or aggregation during thermophoresis. Notably, no aggregation or adsorption was observed for either HOBDI-BF_2_–α-CbTx or eGFP–α-CbTx. The MST experiment was carried out at 40% MST power using a green light emitting diode for excitation. MST data were analyzed and binding parameters were calculated using MO. Affinity Analysis 2 Software (NanoTemper Technologies, Germany). Data points were fitted to a K_d_ model equation according to built-in software recommendations.

### Fluorescent Labeling of nAChRs in Cell Cultures and Tissue Slices

One day before the transfection, Neuro-2a cells were applied to coverslips and placed inside 35-mm cell culture dishes with 2 ml of Eagle’s medium. Transfection medium contained cDNA of human α7 nAChR (3 mg/ml) and the Lipofectamine 2000 transfection reagent (Invitrogen, Thermo Fisher Scientific). The cDNA-containing solution was replaced by cell culture medium 6–8 h after the transfection.

Cryostat 10 μm-thick sections of rat tongue were fixed with isopropanol for 10 min at 4°C, rinsed with PBS and distilled water, air dried for 1 h (all further procedures were carried out at room temperature) and incubated for 1 h with PBS, pH 7.4, containing 10 mg/ml BSA and 5 ml/L Tween 20 to block unspecific binding of toxins. The sections were pre-incubated for 1–2 h in buffer A (1 mg/ml BSA and 150 mM NaCl in PBS). Controls were run simultaneously by adding 300-fold excess of unlabeled α-CbTx (unless specified otherwise). Then, AF555–α-BgTx was added to the slides to reach 50 nM final concentration and they were incubated for 1–15 h. Sections were subsequently washed with PBS, fixed with 40 mg/ml paraformaldehyde for 10 min, rinsed with PBS again and coverslipped in carbonate-buffered glycerol at pH 8.6. The slides were analyzed using CellA Imaging Software (Olympus Soft Imaging Solutions, Germany) coupled to epifluorescent microscope with cooled CCD CAM-XM10 (Olympus, Japan).

### Flow Cytometry

For differentiation of THP-1 into macrophages (THP-1 Mϕ), cells were seeded in 24-well plates and treated with 100 nM phorbol 12-myristate 13-acetate (Sigma-Aldrich) for 48 h. Сells were stained as previously described (Siniavin et al., 2020). Briefly, THP-1 Mϕ, Raji, or RPMI 1788 cells were incubated with 100 nM HOBDI-BF_2_–α-CbTx, eGFP–α-CbTx, eGFP, or AF647–α-BgTx (1 h, at 4°C). Cells were then washed two times with PBS and assayed using BD FACSCalibur or MACSQuant Analyzer 10 flow cytometer (Miltenyi Biotec, Germany) and analyzed using FlowJo software 10.0.8. To determine non-specific binding, cells were pre-incubated with 10 µM α-CbTx before fluorescent toxin staining.

### 3D Model of eGFP–α-CbTx

A model of eGFP–α-CbTx was constructed using UCSF Chimera 1.10.1 software (Pettersen et al., 2004) for Modeller 9.14 (Webb and Sali, 2016). We utilized eGFP (PDB ID: 2Y0G) and α-CbTx (PDB ID: 1CTX) spatial structures as templates. The N-terminal site of chimeric protein and the (G_4_S)_3_-linker between the eGFP and α-CbTx modules were represented in a disordered conformation. A model of eGFP–α-CbTx was visualized by the PyMOL Molecular Graphics System, Version 1.7.4 Schrödinger, LLC.

## Results

### Design and Production of Chimeric eGFP–α-CbTx

**FIGURE 1:**
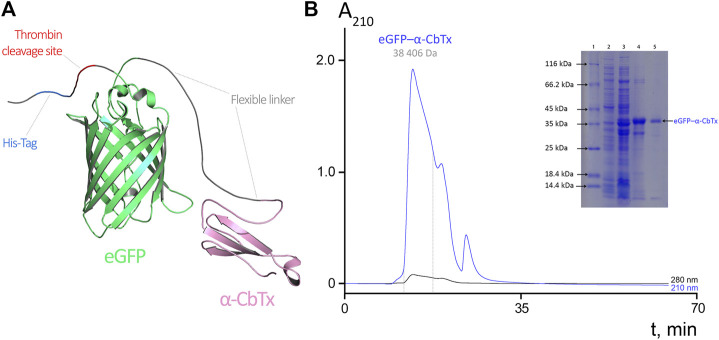
**(A)** Molecular model of eGFP–α-CbTx chimeric protein. eGFP and α-CbTx modules are connected by a short flexible linker. The His-Tag and adjacent thrombin cleavage site are located at the N-terminus of the chimera. **(B)** Size-exclusion chromatography of eGFP–α-CbTx. The dotted lines mark the area corresponding to the collected fraction that contained the chimeric protein. Detection was performed at 210 and 280 nm. The inset shows electrophoretic separation of fractions obtained during the purification of eGFP–α-CbTx in 12% polyacrylamide gel. 1, standards (Pierce Unstained Protein MW Marker, #26610, Thermo Fisher Scientific) for protein electrophoresis; 2, soluble fraction of total cellular protein before induction of expression; 3, after induction by IPTG; 4, chimeric protein after metal-chelate affinity chromatography; 5, chimeric protein after size-exclusion chromatography.

We designed an expression cassette encoding a three-finger toxin fused with a fluorescent protein via a flexible linker (Figure 1A). The well-studied α-CbTx (UniProt ID: P01391) from the venom of *N. kaouthia* (Karlsson et al., 1972) was selected because this protein is highly active towards several targets including muscle-type, neuronal α7 and α9/α10 nAChRs (Chandna et al., 2019), as well as AChBP from *L. stagnalis* (Hansen et al., 2002); α-CbTx binds to these targets with sub-nanomolar affinities. Additionally, it was shown that α-CbTx is able to inhibit ionotropic γ-aminobutyric acid receptors (GABA_A_) at sub-micromolar concentrations (Kudryavtsev et al., 2015). As a fluorescent module, we selected one of the best-studied fluorescent proteins eGFP (FPbase ID: R9NL8) (Cormack et al., 1996) which is characterized by high brightness and weak dimerization properties.

The eGFP–α-CbTx chimera was produced recombinantly, isolated from *E. coli* lysate by affinity chromatography, and purified by size-exclusion chromatography (Figure 1B). Fluorescence excitation and emission spectra of this hybrid were found identical to those of native eGFP protein (excitation and emission maxima were at 488 and 507 nm, respectively). This fact indicates that the spectral properties of the fluorescent protein did not change when it was fused with α-CbTx. The yield of recombinant eGFP–α-CbTx was ∼10 mg per 1 L of bacterial culture; the purity of the final product was not less than 95%.

### Modification of α-CbTx and Aze with *p-*HOBDI-BF_2_

**FIGURE 2:**
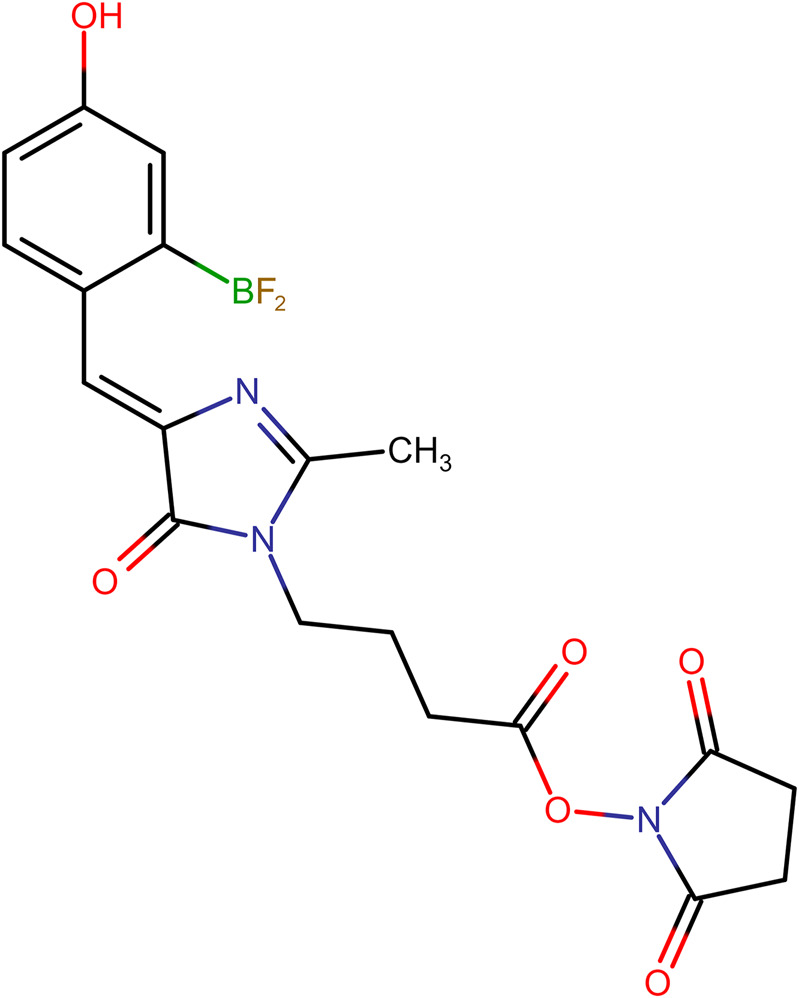
Chemical structure of (Z)-2,5-dioxopyrrolidin-1-yl 4-(4-(2-(difluoroboryl)-4-hydroxybenzylidene)-2-methyl-5-oxo-4,5-dihydro-1H-imidazol-1-yl)butanoate (*p*-HOBDI-BF_2_-OSu).

To obtain *p*-HOBDI-BF_2_-labeled toxins, they were reacted with *p*-HOBDI-BF_2_-OSu (Figure 2) using conditions elaborated earlier for α-CbTx modification by activated esters of photoactivatable labels. The modified α-CbTx was purified using high-performance ion-exchange and reversed-phase chromatography as described earlier (Utkin et al., 1998), and similarly one predominant derivative (HOBDI-BF_2_–α-CbTx) was obtained. This derivative was analyzed by high-resolution mass spectrometry, which showed that it possessed a molecular mass of 8,112.76 Da. This value was 19.92 Da lower than the theoretical mass; the difference is explained by the loss of HF from fluorine-containing label under mass spectroscopy conditions, this phenomenon was earlier described for the BODIPY label (Qi et al., 2015). Peptide mass fingerprinting using trypsin digestion revealed the labeled fragment 13–33 (data not shown), which indicates that α-CbTx derivative contains the label at Lys23.

**FIGURE 3:**
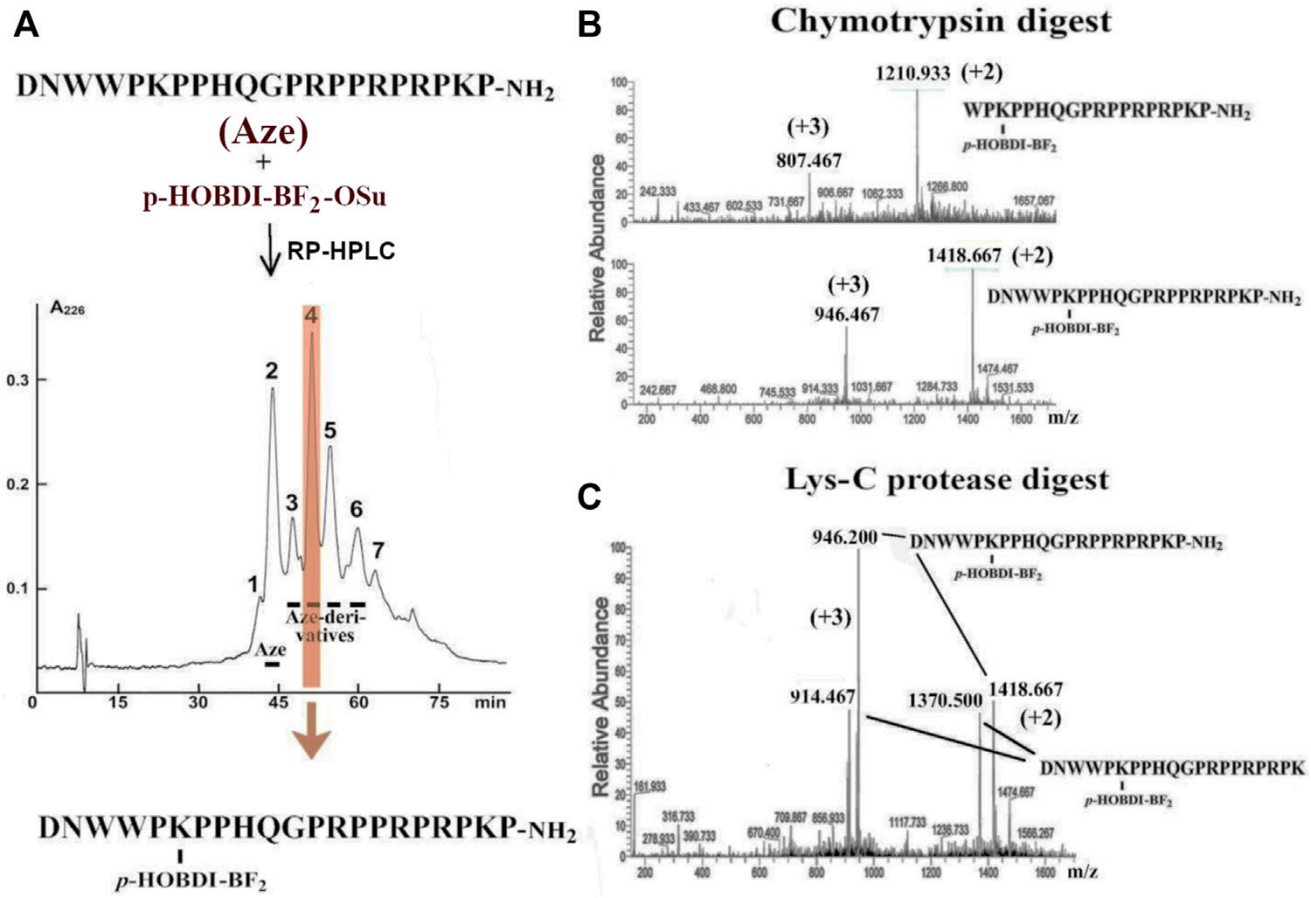
**(A)** Preparation of Aze derivatives. Aze was reacted with *p*-HOBDI-BF_2_-OSu and the obtained derivatives were separated by reversed-phase HPLC on a Jupiter C_18_ column (10 × 250 mm) in a linear gradient of acetonitrile (15–30% in 75 min). Peak 4 (shaded in orange) was found as the best purified individual mono-derivative (HOBDI-BF_2_–Aze) with localization of the photolabel at the ε-amino group of Lys6, as established by chromato-mass spectrometry of its fragments after digestion with chymotrypsin or Lys-C proteinase. **(B)** Chromato-mass spectrometry analysis of peak 4 digested with chymotrypsin revealed the fragments with the measured m/z values 1,418.667 (+2) and 1,210.933 (+2) or 946.467 (+3) and 807.467 (+3) Da. They correspond to the masses of the non-digested HOBDI-BF_2_–Aze and the fragment Aze (4–21) with *p*-HOBDI-BF_2_ label. **(C)** Chromato-mass spectrometry analysis of peak 4 digested with Lys-C protease. Identified m/z values 1,418.667 (+2) and 1,370.5 (+2), or 946.2 (+3) and 914.487 (+3) Da, correspond to the non-digested HOBDI-BF_2_–Aze and the reaction product Aze(1–20) with *p*-HOBDI-BF_2_ label.

A similar technique was applied to introduce the *p*-HOBDI-BF_2_ label into the molecule of amidated Aze (see structure in Figure 3A). The Aze derivatives were purified by reversed-phase HPLC (Figure 3A) and analyzed by mass spectrometry. It was found that fraction 2 with the molecular mass of 2,539 Da represents unmodified toxin. Fraction 3 contains a di-labeled derivative (3,132 Da) contaminated with unmodified toxin. Fractions 4–6 contain different mono-modified products (HOBDI-BF_2_–Aze) with molecular masses of 2,836 Da, fraction 6 being contaminated with a di-labeled product. Interestingly, the mass corresponding to the loss of HF was also observed. As there are 3 amino groups (N-terminal and ε-amino groups of Lys6 and Lys20) in Aze, our results show that three corresponding mono-labeled derivatives were obtained.

According to chromato-mass spectrometry analysis, among all these fractions only peak 4 was an individual compound (data not shown). To localize the fluorophore in this mono-derivative, it was digested with two proteases, and the obtained fragments were analyzed by mass spectrometry. The digestion of this derivative with chymotrypsin resulted in a fragment with molecular mass of 2,420 Da containing the fluorescent label (Figure 3B). This product corresponds to the C-terminal fragment 4–21 of HOBDI-BF_2_–Aze, which indicates that the N-terminal amino group is not modified. To find out which of the two lysine residues (Lys6 or Lys20) is modified, the derivative was digested with Lys-C endoproteinase, and the obtained products were analyzed by mass spectrometry. A peptide with molecular mass of 2,740 Da was found (Figure 3C). It corresponds to the N-terminal fragment 1–20 containing the fluorescent label, suggesting that the fluorophore is attached to Lys6 (see structure in Figure 3A).

### Evaluation of Fluorescent Toxin Affinity to nAChRs and AChBP

#### Competitive Radioligand Assay

**FIGURE 4:**
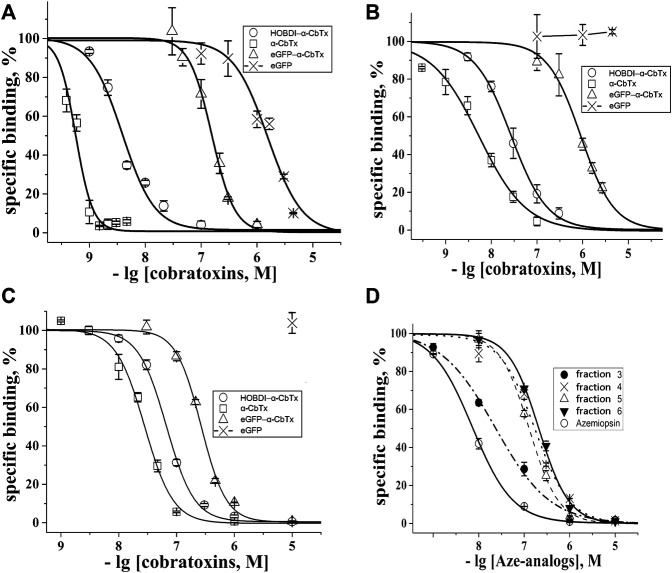
Inhibition of the initial rate for ^125^I-α-BgTx binding to **(A)** *T. californica* nAChR, **(B)** human α7 nAChR and **(C)** *L. stagnalis* AChBP with α-CbTx and its fluorescent analogs (eGFP–α-CbTx, HOBDI-BF_2_–α-CbTx) as well as eGFP. **(D)** Inhibition of initial rate for ^125^I-α-BgTx binding to *T. californica* nAChR with Aze and its *p*-HOBDI-BF_2_ derivatives from fractions 3–6 (see Figure 3A). Each point is a mean ± S.E.M. value of three measurements for each concentration. The curves were calculated from the means ± S.E.M. using OriginPro 2015, and the respective IC_50_ values in nM are presented in Table 2.

We evaluated the affinity of the prepared fluorescent α-CbTx derivatives to the muscle-type nAChR from *T. californica* ray electric organ and neuronal human α7 nAChR expressed in GH_4_C_1_ cells, as well as to AChBP from *L. stagnalis*, which is a homolog of the ligand-binding domain of all nAChRs. This was done by measuring their ability to compete with ^125^I-α-BgTx used as a radioligand. When testing fluorescent α-CbTx (eGFP–α-CbTx and HOBDI-BF_2_–α-CbTx), α-CbTx and eGFP were also used in parallel experiments. The respective inhibition curves on muscle-type and α7 nAChRs, and AChBP are shown in Figures 4A–C and corresponding IC_50_ values and slopes (Hill coefficients) are presented in Table 2.

**TABLE 2:** Affinity of the fluorescent toxins tested in competition with^125^I-α-BgTx for binding to muscle-type nAChR from *T. californica*, human neuronal α7 nAChR, and AChBP from *L. stagnalis* calculated from the respective inhibition curves in Figure 4 as the IC_50_ values and Hill coefficients (n_H_) using OriginPro 2015.

Toxin	*T. californica* nAChR	Human α7 nAChR	*L. stagnalis* AChBP
	IC_50_, nM		
HOBDI-BF_2_–α-CbTx	3.88 ± 0.23 (1.50 ± 0.12)	27 ± 1 (1.10 ± 0.02)	67 ± 2 (1.80 ± 0.07)
eGFP–α-CbTx	155 ± 5 (2.10 ± 0.10)	912 ± 36 (1.20 ± 0.05)	266 ± 7 (2.01 ± 0.09)
α-CbTx	0.57 ± 0.03 (2.86 ± 0.34)	5.4 ± 0.4 (0.84 ± 0.05)	28 ± 1 (1.76 ± 0.12)
eGFP	1,550 ± 120 (1.41 ± 0.15)	>4,500	>10,000
Azemiopsin	7.3 ± 0.2 (0.97 ± 0.02)	—^a^	—
Fraction 3	23 ± 2 (0.72 ± 0.03)	—	—
Fraction 4	172 ± 12 (1.14 ± 0.09)	—	—
Fraction 5	128 ± 3 (1.35 ± 0.04)	—	—
Fraction 6	210 ± 7 (1.31 ± 0.06)	—	—

a —, *not tested*.

It was found that modification of α-CbTx with eGFP leads to a decrease in affinity to all three targets, whereas the incorporation of the small fluorophore HOBDI-BF_2_ has a significantly weaker effect. When *p*-HOBDI-BF_2_ label was inserted, the affinity of the derivative towards muscle-type and neuronal α7 nAChRs, and AChBP was 3.88 ± 0.23, 27 ± 1 and 67 ± 2 nM, respectively, i.e., it decreased 6.8, 5 and 2.4 times (Figures 4A–C; Table 2). The corresponding IC_50_ values for the chimeric eGFP–α-CbTx were substantially higher and reached 155 ± 5, 912 ± 36, and 266 ± 7 nM (Table 2) demonstrating the decrease in affinity 272, 169 and 9.5 times for muscle-type, neuronal α7 nAChRs, and AChBP, respectively. Interestingly, the eGFP itself showed the ability to compete with ^125^I-α-BgTx for binding to the nAChR from *T. californica* with IC_50_ = 1,550 ± 120 nM (Figure 4A), but when the N-terminal His-tag was removed from eGFP, this inhibitory activity disappeared (data not shown).

In order to figure out the reason for such a noticeable drop in affinity of eGFP–α-CbTx, α-CbTx was cleaved from the chimeric fusion protein eGFP–EK-α-CbTx and purified by reversed-phase HPLC. The affinity of purified recombinant α-CbTx towards muscle-type nAChR was markedly lower than that of the native α-CbTx (IC_50_ value was >10 nM vs 0.57 ± 0.03 nM, respectively). This result suggests that recombinant α-CbTx in the chimeric eGFP–α-CbTx protein does not fold properly.

A competitive radioligand analysis was also used to evaluate the affinity of the obtained fluorescent Aze derivatives from fractions 3–6 (Figure 3A) towards the muscle-type *T. californica* nAChR. The inhibition curves and the corresponding IC_50_ values are shown in Figure 4D and Table 2. The affinity of the derivatives from fractions 4–6 decreased after modification by more than an order of magnitude (IC_50_ of 170 ± 50 nM compared to IC_50_ = 7.3 ± 0.2 nM for unmodified Aze). Fraction 3 showed a markedly higher affinity (IC_50_ = 23 ± 2 nM), most likely due to the presence of an unmodified toxin detected by mass spectrometric analysis (see above). The small differences in affinity of mono-labeled derivatives from fractions 4–6 probably reflect the similar importance of modified amino groups for binding to the receptor.

#### Microscale Thermophoresis

**FIGURE 5:**
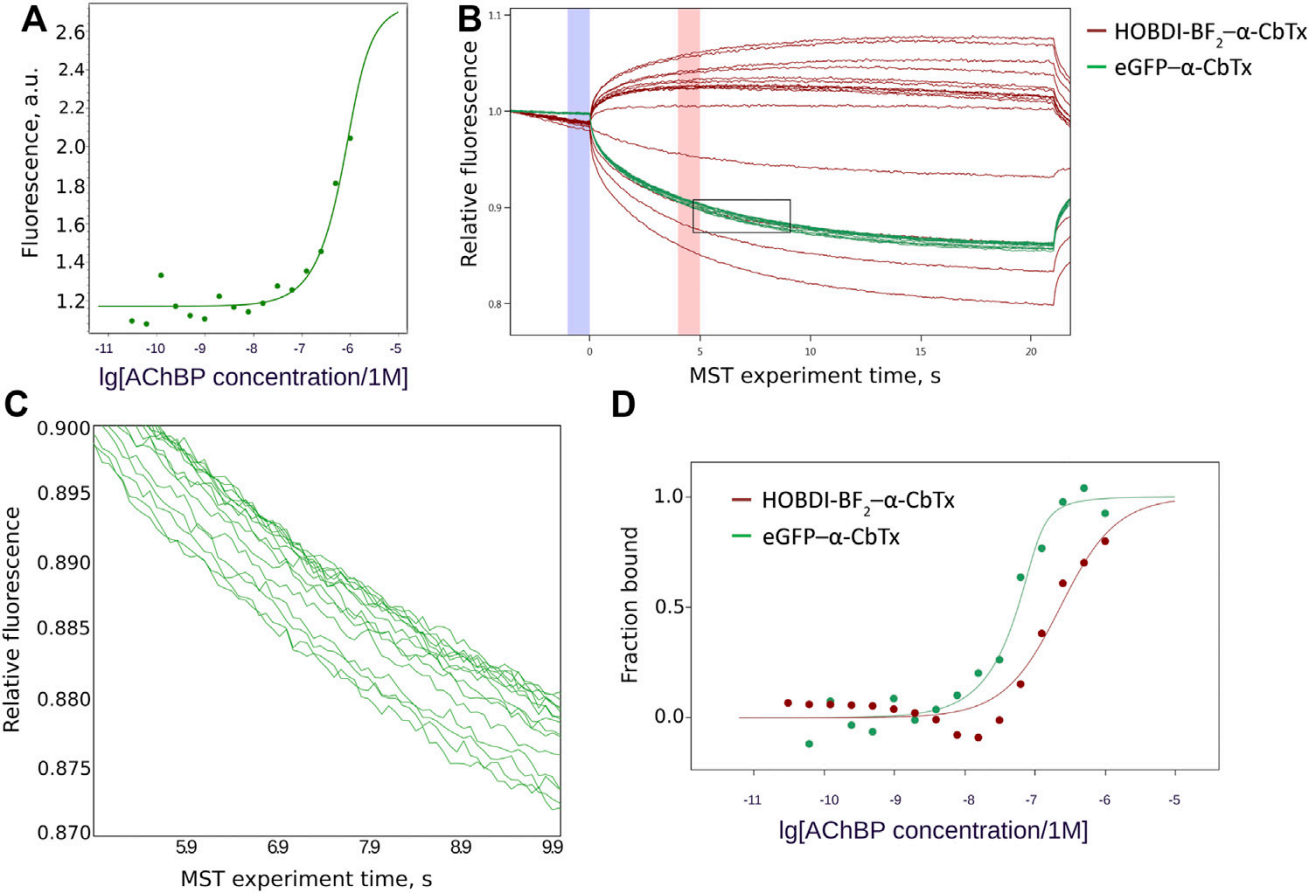
HOBDI-BF_2_–α-CbTx and eGFP–α-CbTx binding to *L. stagnalis* AChBP. **(A)** Changes in initial fluorescence intensity of HOBDI-BF_2_–α-CbTx dependent on AChBP concentration. **(B)** MST traces showing the change of fluorescence over time during the MST experiment. For both HOBDI-BF_2_–α-CbTx (red) and eGFP–α-CbTx (green) a set of MST curves was obtained, each representing a single MST measurement for every concentration of *L. stagnalis* AChBP. Binding curves were calculated from fluorescence values at 5 s (red column on the graph) corrected for fluorescence at 0 time point (blue column on the graph). The region of eGFP–α-CbTx curves enclosed by a rectangle is shown enlarged in the next panel. **(C)** Enlarged region of eGFP–α-CbTx MST traces showing the signal dependence on AChBP concentration. **(D)** Binding curves of eGFP–α-CbTx (70 nM) and HOBDI-BF_2_–α-CbTx (100 nM) to AChBP (60 pM–1 µM). The bound fraction of fluorescent target dependent on ligand (AChBP) concentration is presented.

Interaction of eGFP–α-CbTx and HOBDI-BF_2_–α-CbTx with *L. stagnalis* AChBP was further characterized using MST. To generate a binding curve, we used 70 nM eGFP–α-CbTx and 100 nM HOBDI-BF_2_–α-CbTx, whereas the concentration of AChBP varied from 60 pM to 1 µM. First, the parameters of MST were adjusted to achieve a substantial initial fluorescence. It was found that eGFP–α-CbTx fluorescence is roughly an order of magnitude brighter than HOBDI-BF_2_–α-CbTx, so the excitation power values of 20% for eGFP–α-CbTx and 80% for HOBDI-BF_2_–α-CbTx were used in the subsequent experiments. Pre-MST scan of the capillaries revealed that HOBDI-BF_2_–α-CbTx fluorescence changes significantly upon binding to AChBP (Figure 5A), which allowed a pre-MST estimation of K_d_ ≈ 200 nM. Such changes in initial fluorescence were not observed for eGFP–α-CbTx. Then, the MST experiment was performed and the resulting data points (Figure 5B) were fitted with a K_d_ model to generate binding curves for eGFP–α-CbTx and HOBDI-BF_2_–α-CbTx (Figure 5D). Dissociation constants were calculated: 7 ± 6 nM for eGFP–α-CbTx and 194 ± 89 nM for HOBDI-BF_2_–α-CbTx. Thus, the MST experiment demonstrated a stronger binding of eGFP–α-CbTx to AChBP compared to HOBDI-BF_2_–α-CbTx.

### Application of the Obtained Fluorescent Toxins for Visualization of Biological Targets

#### Fluorescent Labeling of nAChRs in Cell Cultures and Tissue Slices

**FIGURE 6:**
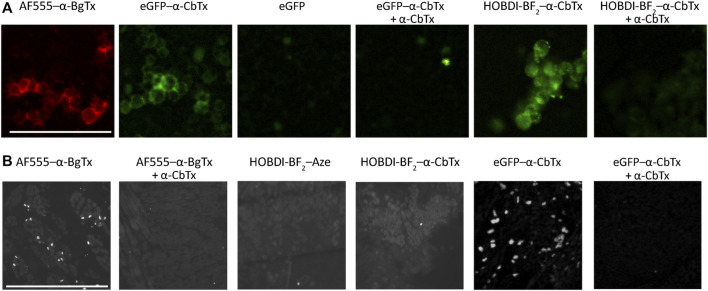
Fluorescence microscopy of Neuro-2a cells transfected with human α7 nAChR, and rat tongue preparation containing muscle fiber end plates with muscle α1β1εδ nAChR. **(A)** Control staining of Neuro-2a cells with 50 nM AF555–α-BgTx shows peripheral staining of the cells, which is reproduced by the eGFP–α-CbTx chimeric protein (500 nM). eGFP alone does not stain the cells. Complementary to that, specific eGFP–α-CbTx staining is abolished by 1 h preincubation with 15 µM α-CbTx. HOBDI-BF_2_–α-CbTx (100 nM) also stains Neuro-2a cells, and it is prevented by 1 h pre-treatment using 5 µM α-CbTx. Scale bar in the first panel, 50 μm. **(B)** AF555–α-BgTx (100 nM) specifically stains end plates in rat tongue cross-sections, and no staining is observed in cross-sections pre-treated with 5 µM α-CbTx. Neither HOBDI-BF_2_–α-CbTx, nor HOBDI-BF_2_–Aze produce end plate staining, whereas 500 nM eGFP–α-CbTx chimeric protein (12 h of incubation) stained cross-sections brightly. End-plate staining by eGFP–α-CbTx chimeric protein is prevented by 1 h pre-treatment with 15 µM α-CbTx. Scale bar in the first panel, 50 μm.

Fluorescence microscopy of Neuro-2a cells transfected with human α7 nAChR, and rat tongue preparation containing muscle fiber end plates with muscle α1β1εδ nAChR, revealed a difference in applicability of the HOBDI-BF_2_ and eGFP derivatives of α-CbTx. Control commercial AF555–α-BgTx (50 nM) shows bright staining of Neuro-2a cells and rat tongue end plates (Figure 6A). Both eGFP–α-CbTx chimeric protein (500 nM) and HOBDI-BF_2_–α-CbTx (100 nM) bind to Neuro-2a cells transfected with human α7 nAChR. This staining is abolished by 1 h preincubation with a 30–50 molar excess of α-CbTx, confirming the specificity of binding (Figure 6A). eGFP alone does not stain cells (Figure 6A), thus confirming that the staining could not be attributed to a non-specific uptake of the fluorescent protein.

eGFP–α-CbTx stained rat tongue end plates brightly and specifically (Figure 6B). Surprisingly, we failed to achieve end-plate staining with HOBDI-BF_2_–α-CbTx. We also tried to stain end plates with HOBDI-BF_2_–Aze, however, no end-plate staining was detected in this case either (Figure 6B). It is worth noting that rat tongue preparations are made using denaturating conditions (isopropyl alcohol). The absence of specific staining of rat tongue cross-sections by the HOBDI-BF_2_ derivatives of neurotoxins could be explained by the intrinsic properties of this fluorophore preventing it from binding to partially denaturated muscle nAChR. On the other hand, the absence of staining by HOBDI-BF_2_–α-CbTx or HOBDI-BF_2_–Aze could be attributed to their fast wash-out kinetics, which might be due to the modification of lysine side chains crucial to the binding.

#### Flow Cytometry Experiments on Different Human Immune Cells

**FIGURE 7:**
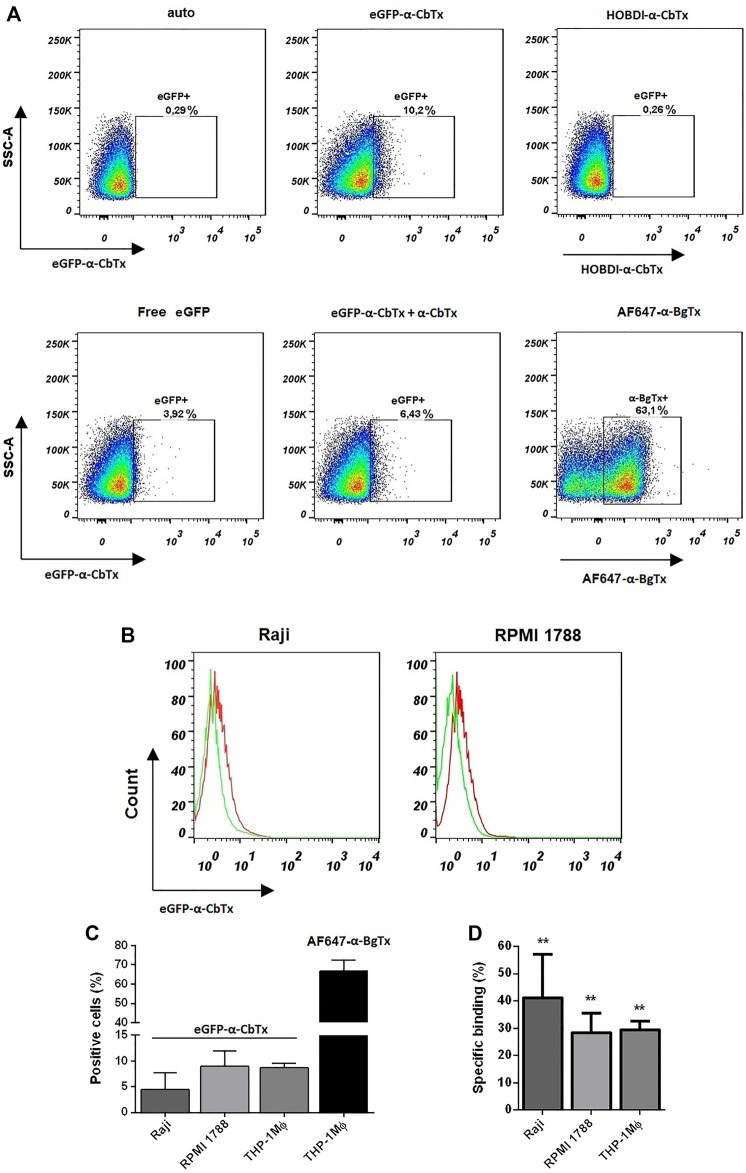
Investigation of cell binding and staining using fluorescent α-CbTx analogs by flow cytometry. **(A)** Representative dot plots of stained THP-1 Mϕ cells using two fluorescent toxins, i.e. eGFP–α-CbTx and HOBDI-BF_2_–α-CbTx. In the top row the autofluorescence, cells stained with eGFP–α-CbTx and HOBDI-BF_2_–α-CbTx, are shown. In the bottom row cells stained with free eGFP, eGFP–α-CbTx with excess of α-CbTx and cells stained with AF647–α-BgTx as a control, are shown. Regions in dot plots represent the percentage of stained cells. **(B)** Representative histograms of Raji or RPMI 1788 cells stained with eGFP–α-CbTx. Red histogram, cells stained with eGFP–α-CbTx; green histogram, cells pre-incubated with an excess of free α-CbTx and then stained with eGFP–α-CbTx. A decrease in the fluorescence of cells due to the binding of free α-CbTx is revealed. **(C)** Diagrams showing the number of positive cells (Raji, RPMI 1788, and THP-1 Mϕ) when stained with eGFP–α-CbTx or AF647–α-BgTx. **(D)** Diagrams of specific binding (%) of eGFP–α-CbTx on different cells from three independent experiments (mean ± S.E.M.)). One-way ANOVA tests with Tukey post hoc test (**p < 0.01) were used.

In flow cytometry experiments, we visualized the binding of eGFP–α-CbTx and HOBDI-BF_2_–α-CbTx to different transformed immune cells, such as Raji, RPMI 1788, and THP-1 Mϕ, expressing α7 nAChR (Figure 7). It was revealed that 100 nM eGFP–α-CbTx stained THP-1 Mϕ cells (Figure 7A). However, the average fraction of stained cells was only 9% (Figure 7C). Pre-incubation of the cells with an excess of free α-CbTx led to a decrease in the percentage of eGFP positive cells (eGFP+) (∼10.2 vs 6.4%; Figure 7A, central panels). Staining of cells with 500 nM eGFP–α-CbTx gave similar results (data not shown). Free eGFP also stained cells (∼3.9% eGFP+; Figure 7A, left panel, bottom row) indicating partial nonspecific binding. In contrast, HOBDI-BF_2_–α-CbTx did not stain the cells, and the level of HOBDI-BF_2_+ cells did not differ from autofluorescence (auto; 0.26 vs 0.29%; Figure 7A, left and right panels, top row). AF647–α-BgTx, which was used as a control, stained THP-1 Mϕ cells ∼7 times better than eGFP–α-CbTx (∼63.1% positive cells; Figure 7A, right panel, bottom row, and Figure 7C).

Next, we performed binding assays using eGFP–α-CbTx to measure the surface expression of α7 nAChR levels on Raji and RPMI 1788 cells. Figure 7B shows that eGFP–α-CbTx can stain both cell lines (red histograms), and this binding is specific because it decreased after pre-incubation with an excess of free α-CbTx (green histograms). However, again the calculated average values for Raji and RPMI 1788 cells labeled with fluorescent toxin (positive cells) were only ∼5 and 9%, respectively (Figure 7C). The average values of specific binding of eGFP–α-CbTx for Raji, RPMI 1788, and THP-1 Mϕ cells were 41, 28, and 30%, respectively (Figure 7D). Flow cytometry measurements showed that eGFP–α-CbTx specifically stains cell lines that express α7 nAChR (Figure 7C). On the other hand, HOBDI-BF_2_–α-CbTx was found unsuitable for this purpose.

## Discussion

Studies of ligand-receptor interactions require adequate molecular instruments and methods. Radioligand analysis was the method of choice for such studies for a long time. However, this method has limitations related to safety and ecological aspects. Fluorescence-based methods became more convenient and popular due to the development of new approaches including super-resolution techniques such as Photoactivated Localization Microscopy (PALM) and Stochastic Optical Resolution Microscopy (STORM) and highly fluorescent labels (e.g., quantum dots) (see reviews (Baddeley and Bewersdorf, 2018; Himmelstoß and Hirsch, 2019)). Among the developed methodologies is the application of fluorescent proteins (GFP and analogs) for the labeling. Modern fluorescent proteins cover a wide range of excitation and emission wavelengths and possess high quantum yields (Chudakov et al., 2010). Recently, we proposed a novel approach for toxin labeling, which is based on the production of chimeric proteins consisting of two modules: a fluorescent protein and a polypeptide toxin specific for certain channels where the ligand part serves for selective channel recognition, and the fluorescent part is used for effective detection. Chimeric fusion proteins comprising GFP (or analogs) and the protein ligand under the study can be easily produced recombinantly in high quantities. Relying on this approach we produced a chimera (eGFP-OSK1) for potassium channel recognition (Kuzmenkov et al., 2016a). Further, this approach was also applied for K_V_1.3-specific chimera production due to clinical importance of this isoform (Nekrasova et al., 2020).

The preparation of fluorescently labeled proteins and peptides by chemical modification is another frequently used method for which appropriate reagents have long been developed. N-Hydroxysuccinimide esters of respective fluorophores are most often used, resulting in modification of amino groups (the N-terminal α-amino group or ε-amino groups of lysine residues). This method allows introducing labels into different parts of the molecule, which is not always possible with fluorescent proteins. However, sometimes the introduction of large fluorophores into peptides or small proteins can lead to a loss of activity. Nevertheless, fluorescently labeled toxins were successfully used to study toxin-receptor interactions. We were among the first in application of fluorescently labeled TFTs for the investigation of nAChRs (Tsetlin et al., 1979, 1982; Surin et al., 1983) and we continue to successfully use them for this purpose until now (Shelukhina et al., 2009; Pivovarov et al., 2020). The first studies showed the preferential modification of the lysine in the central loop of TFTs, which did not lead to a significant change in the affinity to the receptor, and allowed to characterize the toxin site involved in the interaction with nAChR.

When introducing bulk moieties into small peptides, a noticeable decrease in affinity, a change in specificity or an increase in non-specific interactions is more often observed, as we demonstrated earlier with the example of some photoactivated derivatives of α-conotoxins MI and GI (Kasheverov et al., 2001, 2006). However, fluorescent derivatives of some α-conotoxins were prepared, which became selective markers of different nAChR subtypes (Hone et al., 2010; Surin et al., 2012; Barloscio et al., 2017). For instance, using a fluorescent derivative of α-conotoxin MII, a selective α6β2 nAChR antagonist, α6-containing nAChRs were localized in the retinal tissue and found to be predominantly localized to the ganglion cell layer (Barloscio et al., 2017).

The introduction of a bulky fluorescent label can result in a decrease of affinity and change of selectivity of toxins. However cryo-EM structure of *Torpedo* acetylcholine receptor in complex with α-bungarotoxin (Rahman et al., 2020) reveals that both N- and C-termini of TFTs are less important for the interaction with the receptor and can tolerate the attachment of a sizeable substituent. Keeping this in mind we prepared a chimeric protein, in which eGFP was fused with α-CbTx N-terminus through a linker of 19 amino acid residues (Figure 1). In radioligand assays, the chimeric protein with a full-sized GFP (eGFP–α-CbTx) showed a significantly diminished affinity towards all tested targets as compared to α-CbTx. The decrease in affinity for muscle-type *T. californica*, human α7 nAChR, and *L. stagnalis* AChBP was ∼272, 169 and 9.5 times (Figures 4A–C; Table 2). To find out the reasons for this discrepancy, the toxin was split from eGFP and purified. The obtained recombinant α-CbTx was substantially less active than the native toxin, leading us to the assumption that an essential part of the recombinant toxin was incorrectly folded.

As mentioned in the Introduction, the synthetic analog of GFP chromophore *p*-HOBDI-BF_2_ was prepared earlier (Baranov et al., 2012). This label is similar in many of its spectral characteristics to the well-known dye fluorescein. It has a similar spectrum, close extinction coefficient, and quantum yield of fluorescence. However, compared to fluorescein, HOBDI-BF_2_ is minute, its molecular mass is 4 times smaller. It is also assumed as more biomimetic since it is based on the GFP chromophore. Thus, the use of this label should affect the properties and behavior of the stained biological objects to a significantly lower degree. To utilize this chromophore for specific amino group labeling in proteins, an activated N-hydroxysuccinimide ester was synthesized. We introduced *p*-HOBDI-BF_2_ into α-CbTx, and one predominant fluorescent derivative HOBDI-BF_2_–α-CbTx was obtained. Compared to native α-CbTx, this derivative showed a reduced affinity to muscle-type *T. californica*, human α7 nAChR, and *L. stagnalis* AChBP by ∼6.8, 5.0 and 2.4 times, respectively (Figures 4A–C; Table 2), still retaining a high affinity to all tested targets. Since α-CbTx binds to both muscle-type and some neuronal nAChRs, we also prepared fluorescent derivatives of Aze, a selective antagonist of muscle-type nAChR. *p*-HOBDI-BF_2_ introduction into Aze led to the formation of multiple reaction products (mono- and di-derivatives with possible label localization at the α-amino group of Asp1 and ε-amino groups of Lys6 or Lys20; Figure 3A). To identify the exact localization of the label for the best purified derivative (fraction 4), a chromato-mass spectrometrу analysis of its chymotrypsin (Figure 3B) and Lys-C fragments (Figure 3C) was performed. Interestingly, the introduction of a *p*-HOBDI-BF_2_ chromophore into a small peptide Aze did not dramatically decrease its affinity to muscle-type nAChR (Figure 4D).

The evaluation of the binding of two fluorescent toxins (eGFP–α-CbTx and HOBDI-BF_2_–α-CbTx) to AChBP using MST (Figure 5) showed that the affinity of the photoprobes lies in the same concentration range, i.e. 10–200 nM. This result suggests that both methods of obtaining fluorescent products (recombinant and chemical modification) used by us lead to sufficiently effective tools for detecting the respective receptor targets.

Optical microscopy is one of the major fields for the application of fluorescent derivatives of neurotoxins. Both HOBDI-BF_2_–α-CbTx and eGFP–α-CbTx were applicable for the detection of nAChR in live cell imaging (Figure 6A). eGFP–α-CbTx is able to detect muscle nAChR in a tissue preparation and represents a cheap analog of the chemically modified α-BgTx conventionally utilized for this task (Figure 6B). However, neither HOBDI-BF_2_–α-CbTx nor HOBDI-BF_2_–Aze were able to stain histological cross-sections of rat tongue containing muscle nAChR. Their inability to bind to nAChRs that are present in rat tongue preparations could be explained by partial denaturation of the receptor due to isopropyl alcohol treatment. As a result of such treatment some fluorescent toxins retain the ability to bind to muscle nAChR (AF555–α-BgTx, eGFP–α-CbTx), but others lose their affinity. These features of new fluorescent derivatives should be taken into account in experimental design.

In flow cytometry experiments (Figure 7), it was revealed that a very small proportion (<10%) of the three studied cell types (Raji, RPMI 1788, and THP-1 Mϕ) expressing α7 nAChR (Siniavin et al., 2020), is specifically stained by eGFP–α-CbTx. The use of HOBDI-BF_2_–α-CbTx produced results at the level of autofluorescence (Figure 7A). This is in particular contrast to the commercially available AF647–α-BgTx (Figures 7A,C), which stained ∼60% of cells. Thus, there are applications where our novel derivatives are efficient, but there are numerous cases where traditional readioactive and fluorescent labels retain their role.

## Data Availability

The raw data supporting the conclusions of this article will be made available by the authors, without undue reservation.
